# Supplementary material for: Evaluation of family planning service delivery in Gondar city public health facilities, Northwest Ethiopia: A cross-sectional study
Source: PLoS One. 2022 Sep 23;17(9):e0274090. doi: 10.1371/journal.pone.0274090 (PMC9506637; doi:10.1371/journal.pone.0274090)
Supplement: S1 File — (PDF) [file pone.0274090.s001.pdf]

English version of data collection tools

Client exit interview tool to assess client's satisfaction on family planning services at Gondar city administration public health facilities, 2020.

Introduction and consent

Hello my name is _____________.I am a data collector to evaluate family planning service. The purpose of this study is to evaluate the implementation status of family planning services provided in public health facilities in Gondar City administration. The evaluation finding is important to strengthen and improve the family planning service. The interview will take about 15 minutes. No information concerning you, as individual will be passed to another individual or health facilities without your permission. Your participation is voluntary and you have the right to withdraw from the study at any time. If you agree to be participate in the study, only honest answers would contribute to improvement of family planning service.

May I continue?

1) Yes, continue interviewing.

2) No, thanks and stop interviewing

Name of the interviewer___________________________________ Sign. ____________

date of interview_______

Name of the supervisor _______________________Sign._______ date________________

Code number of the client __________________Client arrived at service delivery points ________time client received service ____Waiting time_______

Socio-demographic information tools.

Family planning service users interviewer-administered exit questioner.

No.	Questions	choices	Skips
1	Age of the respondent?	_________ years	
2	Educational level of the respondent?	Educational status__________	
3	Religion	1. Orthodox<br>2. Muslim<br>3. Protestant<br>4. Catholic<br>5. Other (specify)___________	
4	Main occupations	1. Unemployed<br>2. Governmental employee<br>3. Merchant<br>4. Housewife<br>5. Daily laborer<br>6. Other (Specify	
5	What is the average household monthly income?	Monthly income in Ethiopian Birr_____________	
6	Marital status	1. Married<br>2. Single<br>3. Widowed<br>4. Divorced<br>5.separated<br>6. Other (specify)________	

7	Residence of the client?	1.Urban<br>2.Rural	
8	Family size of the household?	__________	
9	Did you come to this health facility previously	1. Yes<br>2. No	
10	How can you visit this health facility?	1.Came after referral<br>2.Came due to emergency<br>3. Came upon recommendation from friend/relative<br>4.Came upon personal decision<br>5.Other(specify)	
11	Travel distance from home to health facility in KM?	________________	
12	Waiting time to meet service provider (in minute)	____________	
13	Have you discussed about family planning with your husband?	1.Yes<br>2.No<br>3.Don‘t remember	
14	Would you like to have more children?	1.Yes<br>2.No<br>3.Depends on God<br>4.Depends on husband<br>5.No answer	
15	If yes, when would you like to have the next child?	1.now<br>2.One - two year<br>3.After two years<br>4.No answer	
16	Are you currently lactating mother?	1.Yes<br>2.No	

client perspective satisfaction tools

No.	Questions	Satisfaction scale				
1	The distance and location of the facility is good for me.	1	2	3	4	5
2	The family planning room is clean for me	1	2	3	4	5
3	I am comfortable with the schedule or working hours of family planning clinic	1	2	3	4	5
4	The waiting room is good for me	1	2	3	4	5
5	The counseling room is convenient	1	2	3	4	5
6	I am satisfied with the examination and consultation I have received to day	1	2	3	4	5
7	I am satisfied with the privacy in the service room	1	2	3	4	5
8	The provider explained well about the contraceptive methods	1	2	3	4	5
9	The provider used teaching aids	1	2	3	4	5
10	The facility had functional latrine & piped water	1	2	3	4	5
11	I am satisfied with the overall service that I got today	1	2	3	4	5
12	The provider who gave the service today had adequate skill	1	2	3	4	5
13	I am satisfied with the appointment time	1	2	3	4	5

Thank you for giving your time and cooperation in answering my questions!!

Resource inventory checklist to observe for family planning program service.

Good morning dear!

My name is ---------------------------- I am a member of research team for process evaluation of family planning service, which is going to be conducted by University of Gondar School of public health. The purpose of this study is to evaluate implementation level of family planning service in public health facilities in Gondar city administration. The finding of this study is intended to improve family planning program in the health facilities. The observation includes various techniques to evaluate your interaction. In order to attain effectively the goal of this study, I am asking you for your generous participation. I don't put your name or registration number on this questionnaire. It is your full right to refuse or participate in the study. But your honest response will contribute to generate information, which can be used to improve the implementation status of family planning program.

Do you agree to participate in this study?

1. Yes
2. No

I. General information and human resources

Health institution Name	
Code of health institution	
Date of inventory observation	
Name and signature of observer	
Supervisor name and signature	
Total health care providers in this health facility	
No. of health care providers trained on family planning	

II. Consumable resources

No.	Items	Available at the time of data collection		Available in the last 3 month		Remark
2.1	Disposable gloves	Yes	No	Yes	No	
2.2.	Sterile gloves					
2.3	5 ml syringes with needle					
2.4	Antiseptic solutions (Iodine or Chlorhexidine)					
2.5	Sterile surgical drapes					
2.6	Lidocaine (local anesthesia)					
2.5	Disposal containers for contaminated waste/supplies					
2.6	Clean instrument containers					
2.7	containers for used sharp material disposal					
2.8	Skin bandage or Band-Aid					
2.9	Gauze sterile					
2.10	Dry Cotton					
2.11	Functional mini lap kits (HIV test kit,RBS,)					
2.12	Pregnancy test					
2.13	Soap on hand washing area?					

2.14	Single use towel					
2.15	Decontamination solution					

III. Non-consumable /re-used/ resource

No.	Items	Available at the time of data collection		Remark
		Yes	No	
3.1	Functional Sterilizer (Autoclave) in the HC?			
3.2	Functional Blood pressure apparatus?			
3.3	Stethoscope?			
3.4	Functional Thermometer?			
3.5	Functional Weight Scale?			
3.7	Instrument trays?			
3.8	Examination couch or table?			
3.9	Functional equipment Lamplight?			
3.10	Functional uterine sound?			
3.11	Functional Speculum?			
3.12	Functional Scissors?			
3.13	Functional Temecula?			
3.14	Minor Surgery equipment’s?			

3.15	Job aids (WHO medical eligible criteria wheel)?			
3.16	Posters			
3.17	Flip charts			
3.18	Brochure/pamphlet			
3.19	Anatomical model			
3.20	Information sheet			
3.21	Direction indicator outside the service room			
3.22	FP guide line 2015			
3.23	Recording- log book			
3.24	Integrated card			
3.25	Tally sheets			
3.26	Referral form			
3.27	Reporting form			

IV. **contraceptive**s

No.	Items<br>Does all items contraceptives available in the clinic?	Available at the time of data collection		Available in the last 3 month		Remark
		Yes	No	Yes	No	
4.1	Combined oral contraceptives					
4.2	Progestin-only contraceptives					

4.3	Combined injectable contraceptives					
4.4	Progestin-only injectable contraceptives					
4.5	Male condoms					
4.6	Female condoms					
4.7	Emergency contraceptive pills					
4.8	Implant/Implanon 3 years					
4.9	Implant/Implanon 5 year					
4.10	IUCD					
4.11	Spermicidal					
4.12	Cycle beads for standard days method					
4.13	Male sterilization (Vasectomy)					
4.14	Female sterilization					

V. Infrastructure

No	Items	Available at the time of data collection		Remark
		Yes	No	
5.1	Electricity with backup generator			
5.2	Water			
5.3	Working toilet			
5.4	Telephone			

5.5	Waiting area for clients			
5.6	Examination room			
5.7	Table and seat for service provider			
5.8	Water for hand-washing			
5.9	Soap on hand washing area?			
5.10	Single use towel			
5.11	Decontamination solution			

Checklist to observe the compliance of health care providers during service delivery

Consent form for health care providers:

My name is ________from University of Gondar and I am here to observe the clinical sessions at this unit. This evaluation study will help to improve the implementation of family planning services delivered at this health facility. The observation will be conducted while you deliver services and all findings of the observation will be kept confidentially. Furthermore; we will ensure that any information in our report does not identify you as the respondent.

Are you willing to participate in this observation?

1. Yes 2. No

(If yes I will continue, if No I stop)

_________________ _______________

Observer date

No	For compliance observation chick list	yes	No
1	Dress and have ID based on dressing code of ethics while serving clients?		
2	Demonstrates good counseling skills (asking open-ended questions)?		
3	Assures client of confidentiality during the counseling and clinical session		
4	Asks client about reproductive intentions (more children? when? Or long and short term reproductive intentions of the client.)		
5	Discusses with client which method she would prefer?		
6	Mentions HIV/AIDS (STI) (initiates or responds)		
7	Treats client with respect/courtesy		
8	Service provider tells key information to the particular needs of the specific client		
9	Gives accurate information on the method accepted (how to use, side effects, complications)		
10	Follows infection control procedures outlined in guidelines		
11	Recognizes/identifies contraindication consistent with guidelines		
12	performs clinical procedures according to Guidelines		
13	Client participates actively in discussion and selection of method (is “empowered”)		
14	Offers at least two modern methods of FP		
14	Fill properly the national registration documents		
15	Are the protocol and guidelines used consistently?(observe)		

16	Are facilities for storing contraceptives adequate?		
17	Service provider gives instructions on when to return?		

Key informant interview tools

I am carrying out process evaluation on family planning service in public health facilities in Gondar city administration to find ways of improve the service. I would like to ask you some questions to get information from your experience. Please be sure that this discussion is strictly confidential and that your name is not being recorded.

May I continue?

Yes

No

Health facility name ______________________________

Code of the health facility ___________________________

Code of the key informant_________________________

Position of the key informant___________________________

How long you have been in this position in months________________

Date of interview____________________________________

Interviewer name and signature________________________

I. key informant personal information

Sex _____age _____marital status _____educational status _____________

How long have you been working here? ___________

Questions

1. Is there annual plan to deliver Family planning service?
   a) If yes how you participate the health care provider's in the planning process? __
   b) If no why? ____________
2. Is there internal supportive supervision (ISS) in this health facility in the last six months?
   a) If yes, how frequent? ________
   b) With whom you conducted? _______
   c) If no why? __________________
3. Is there a system to promote family planning service to the community?

a) If yes, please describe how it is conducted? ___________________

b) If no why? _________________________

4. Is there monitoring and evaluation system in Family planning program in the health center?

a) If yes, when and who conduct it? ______________

b) If no, why? ______________________

5. Are there barriers for implementation of family planning service in the health center? a) If yes what are the barriers? ________

6. Are resources for family planning service deliver in place?

a) If yes list the available resources in the health center? _________________

b) If why? _____________________

7. Had the Family planning services interrupted due to unavailability of supplies and drugs in the last 6 months?

a) If yes specify? _________

8. In your opinion, what are the prominent factors affecting Family planning service delivery from health care provider's perspective? _______________

9. What are the most common complaints forwarded by Family planning service utilization? _____________________

10. Do you advertise (promote) FP program in any way to the community? __________

a) If yes describe some of the activities? ________

b) If no why? ______________
