# Supplementary material for: Evaluation of family planning service delivery in Gondar city public health facilities, Northwest Ethiopia: A cross-sectional study
Source: PLoS One. 2022 Sep 23;17(9):e0274090. doi: 10.1371/journal.pone.0274090 (PMC9506637; doi:10.1371/journal.pone.0274090)
Supplement: Attachment — Submitted filename: Point by point responses to reviewers.docx [file pone.0274090.s004.docx]

**Point by point responses to reviewers**

| **S.No** | **Reviewers’ and editor’s comments** | **Authors’ responses** |
| --- | --- | --- |
|  | **Editor’s questions and comments** | Dear editor and reviewers thanks for your constructive comments and suggestions. We have revised the manuscript in detail. All indicated line no. and page no. are on the track change. |
| 1. | The University of Gondar sponsored this evaluation. However, it has no role in the decision to publish, manuscript preparation, and publication.”  We note that you have provided funding information that is not currently declared in your Funding Statement. However, funding information should not appear in the Acknowledgments section or other areas of your manuscript. We will only publish funding information present in the Funding Statement section of the online submission form.  Please remove any funding-related text from the manuscript and let us know how you would like to update your Funding Statement. Currently, your Funding Statement reads as follows:  “The author(s) received no funding for this work.”  Please include your amended statements within your cover letter; we will change the online submission form on your behalf. | Kindly see the funding statement line 421 |
| 2. | “The authors have declared that no competing interest.”  Please complete your Competing Interests on the online submission form to state any Competing Interests. If you have no competing interests, please state ""The authors have declared that no competing interests exist."", as detailed online in our guide for authors at <http://journals.plos.org/plosone/s/submit-now> | Thank you. Kindly see computing interest section line 419 |
| **2** | We note that you have stated that you will provide repository information for your data at acceptance. Should your manuscript be accepted for publication, we will hold it until you provide the relevant accession numbers or DOIs necessary to access your data. If you wish to make changes to your Data Availability statement, please describe these changes in your cover letter and we will update your Data Availability statement to reflect the information you provide. | All the data used are attached with annexes of this document. Interestingly see data availability statement line 417 and supporting information line 522-527 |
| 3. | Please include a separate caption for each figure in your manuscript. | Kindly see in line 527 |
| **Reviewer # 1** | |  |
| 1. | The study is well designed and the work process is carefully described. The results of this study are very valuable for evaluating the family planning program in the area; but it is clear that the results will be specific to the same time and place. | Yes you are right. We put it as a limitation of this study. Kindly see line 380-385 |
| **Reviewer # 2** | |  |
| 1. | Please describe more clearly about the analysis of qualitative study in the method section. | It is well described at data management and analysis section; line 199-203 |
| 2. | Please check table 4–socio-demographic characteristics of clients using family planning services in Gondar City. The total frequency of each variable should be the same number. If it is different, please explain it in the method section. | We revised the tables (Table 4), line 283-285 |
| 3. | Please add more transcript of qualitative study. In the study, there are only 3 transcripts of the interviews | Kindly see line 222-225,242-252 and 266-251 |
| 4. | What is star (*) for in the table 6, please add in the note under the table | *Independent Variables having significant association with outcome variable in multi-variable regression analysis (kindly see in line 309) |
| 5. | Table 6: why do you keep including variables that are not significant in bivariate analysis into multivariate analysis? | Thank you for your comment! We revised the table. kindly see table 6 |
| 6. | Please add the recommendation related to the contraceptive stocked out in the health facilities in the past three months | Kindly see line 387-299 |
| **Reviewer # 3** | |  |
| 1. | Abstract-- The results reported in the abstract is limited in comparison to what was detailed in the methods section of the abstract. | Thank you for your comment; the abstract is revised. Kindly see line 18-46 |
| 2. | Introduction – The introduction as is written is sometimes unclear to me, and the aim of the study appears vague. I suggest re-writing aspects of the introduction to improve clarity of the background behind the study and to clearly state the study aim/objective. | See the introduction section line 48-84 |
| 3. | Sampling: Can the authors provide details on what the sample distribution was within the eight family planning service facilities. i.e. did most of the sample come from just one of the facilities or was it evenly spread? | Thank for your comment, we have revised it. For detail kindly see it in line |
| 4. | Variables (lines 149 to 155): Can the authors describe why they sought these independent variables? Based on experience or literature? Also the authors should state how they collected data for these variables – patient reported, interview or structured interview questionnaire? | Kindly see in line 249-254 |
| 5. | Can the authors please name and describe the validated tools that were adapted or used for assessing ‘availability of resources’, ‘Compliance’, and ‘Acceptability’? | The sources for these validated tools were cited. kindly see in variables and measurement section : line 157-183 |
| 6. | Can you clarify what you mean by ‘formative approach’ on line 123 | It is revised in line119-121 |
| 7. | Can you clarify what you mean by ‘exit interview’ and ‘key informant interview’ on line 145, including which study participants where sampled for the respective interviews (i.e service users, program managers, healthcare workers etc.) | It is revised in line 141-146 |
| 8. | In Table 3,5,& 7 the ‘Expected’ column is blank | Kindly see the revised tables |
| 9. | I suggest the authors consider reviewing the title of their study to something along the lines, 'An evaluation of family planning service delivery in Gondar city public health facilities Northwest Ethiopia; a cross-sectional study' | Thank you for your comment and the title is modified based on your suggestion |
| 10. | Study dates don’t match up can you clarify. In the abstract you report ‘March 10 to April 25, 2020’ and on line 113 you report ‘March 25 to April 25, 2020’. | Thank you for your view; it was type error and it is corrected in abstract and methods section |
| 11. | I am not sure ‘predictors’ (line 28) of an outcome can be assessed from a cross-sectional study. I suggest the authors rephrase this to ‘factors associated with patient satisfaction’ | Thank for your suggestion; we revised it in line 27-28 |
| 12. | Manuscript needs proofing to correct some grammatical errors. For example in lines 54-56, lines 174, lines 223-225 | The manuscript is revised: kindly see line 61-63,185-191, and 228-231 |
| 13. | Please define what EDHS stands for on line 100. | It is defined in line 95 |
| 14. | Also the authors had 100% response rate for the service user interviews. Does this mean all service users approached agreed to participate in the study? | Of course; all service users asked to participate in the interview were agreed to respond the questionnaires. |
| 15. | PLOS authors have the option to publish the peer review history of their article ([what does this mean?](https://journals.plos.org/plosone/s/editorial-and-peer-review-process#loc-peer-review-history)). If published, this will include your full peer review and any attached files. If you choose “no”, your identity will remain anonymous but your review may still be made public | Thank you for your comment; we revised it in this amendment. |
